# Toward Developing Chemical Modulators of Hsp60 as Potential Therapeutics

**DOI:** 10.3389/fmolb.2018.00035

**Published:** 2018-04-20

**Authors:** Qianli Meng, Bingbing X. Li, Xiangshu Xiao

**Affiliations:** Program in Chemical Biology, Department of Physiology and Pharmacology, Oregon Health and Science University, Portland, OR, United States

**Keywords:** autoimmune, cancer, chaperone, GroEL, GroES, Hsp60, Hsp10, inhibitor

## Abstract

The 60 kDa heat shock protein (Hsp60) is classically known as a mitochondrial chaperonin protein working together with co-chaperonin 10 kDa heat shock protein (Hsp10). This chaperonin complex is essential for folding proteins newly imported into mitochondria. However, Hsp60, and/or Hsp10 have also been shown to reside in other subcellular compartments including extracellular space, cytosol, and nucleus. The proteins in these extra-mitochondrial compartments may possess a wide range of functions dependent or independent of its chaperoning activity. But the mechanistic details remain unknown. Mutations in *Hsp60* gene have been shown to be associated with neurodegenerative disorders. Abnormality in expression level and/or subcellular localization have also been detected from different diseased tissues including inflammatory diseases and various cancers. Therefore, there is a strong interest in developing small molecule modulators of Hsp60. Most of the reported inhibitors were discovered through various chemoproteomics strategies. In this review, we will describe the recent progress in this area with reported inhibitors from both natural products and synthetic compounds. The former includes mizoribine, epolactaene, myrtucommulone, stephacidin B, and avrainvillamide while the latter includes *o*-carboranylphenoxyacetanilides and gold (III) porphyrins. The potencies of the known inhibitors range from low micromolar to millimolar concentrations. The potential applications of these inhibitors include anti-cancer, anti-inflammatory diseases, and anti-autoimmune diseases.

## Introduction

Anfinsen's pioneering experiments demonstrated that the primary amino acid sequences of small proteins will dictate their final native conformations (Anfinsen, 1973). For larger proteins, however, molecular chaperones are needed in the cells to help achieve their native conformations (Bukau and Horwich, 1998; Finka et al., 2016). The human 60 kDa heat shock protein 60 (Hsp60), which is also known as 60 kDa chaperonin (Cpn60) and was initially cloned by Jindal et al. (1989), is the homolog of bacterial GroEL (Hemmingsen et al., 1988). GroEL, in conjunction with cochaperonin GroES is the major molecular chaperone in bacteria to help unfolded and/or partially folded polypeptides fold into their native conformations. Increasing evidence has also shown that the GroEL-GroES complex plays a critical role in partial unfolding of misfolded intermediates for further folding (Shtilerman et al., 1999; Lin et al., 2008; Weaver et al., 2017). Structural studies have shown that the GroEL-GroES complex undergoes extensive conformational changes during the folding pathway wherein the hydrophobic patches can initially bind unfolded polypeptides primarily through hydrophobic interactions (Finka et al., 2016). Large conformational changes in GroEL help fold the hydrophobic residues in the substrates into protein interior to facilitate folding. The conformational changes of GroEL are driven by multiple factors including GroES binding, substrate binding, ATP binding and ATP hydrolysis (Horwich and Fenton, 2009).

Hsp60 was initially characterized as a nuclear-encoded mitochondrial protein to help fold proteins newly imported into mitochondria in conjunction with co-chaperonin Hsp10 (10 kDa heat shock protein) (Jindal et al., 1989; Ostermann et al., 1989; Reading et al., 1989). Interestingly, anti-folding activity of Hsp60 has also been reported for certain substrates (e.g., cytochrome b_2_) shortly after the discovery of Hsp60 as a molecular chaperone (Koll et al., 1992). While Hsp60 was thought to be only localized in mitochondria, accumulating data support that it is localized in extramitochondrial compartments as well. These include cytosol (Soltys and Gupta, 1996; Kirchhoff et al., 2002; Chun et al., 2010; Campanella et al., 2012; Kalderon et al., 2015), outer mitochondrial surface (Soltys and Gupta, 1996), cell surface (Soltys and Gupta, 1996, 1997; Piselli et al., 2000; Feng et al., 2002), intracellular vesicles (Soltys and Gupta, 1996), nucleus (Itoh et al., 1995), extracellular space (Soltys and Gupta, 1996; Gupta and Knowlton, 2007), and even in blood circulation (Pockley et al., 1999; Lewthwaite et al., 2002; Shamaei-Tousi et al., 2007; Hamelin et al., 2011). While the function of Hsp60 in these extra-mitochondrial compartments might also involve its chaperoning activity, it is unlikely that its functions in these different locations can be explained solely by its chaperoning activity. Therefore, Hsp60 can be considered as a protein with moonlighting functions (Henderson et al., 2013). In this article, we review the development of chemical modulators of Hsp60 as potential therapeutics. We will primarily focus on the mammalian Hsp60 whereas the bacterial counterpart will be provided as necessary background and comparison purposes.

## Hsp60 and human diseases

As a mitochondrial chaperone, Hsp60 is essential for mitochondrial protein homeostasis (Cheng et al., 1989; Ostermann et al., 1989). The significance of Hsp60 in humans is further illustrated by many disease-associated mutations in *Hsp60* (also called *HSPD1*) (Hansen et al., 2002, 2007; Parnas et al., 2009; Christensen et al., 2010; Bross and Fernandez-Guerra, 2016). For example, V98I mutation in Hsp60 was reported to be associated with hereditary spastic paraplegia SPG13, a rare neurodegenerative disorder characterized by spasticity and weakness of the lower limbs (Hansen et al., 2002; Bross et al., 2008). No effective treatment for SPG13 exists. Biochemically, this mutation is accompanied with reduced capacity in refolding Hsp60 client proteins (Bross et al., 2008). Another mutation in Hsp60 (D27G or D3G in the mature form) was identified from a large kindred with 10 patients suffering from MitCHAP-60 disease, which is an autosomal-recessive neurodegenerative disorder (Magen et al., 2008). This debilitating early onset disease is characterized by hypomyelination and leukodystrophy in the brain. In an attempt to understand the mechanism by which this mutation contributes to the disease, it was found that the D3G mutant was less stable in forming heptameric and tetradecameric oligomers than wild type. This is further accompanied with decreased refolding capacity and ATPase activity (Parnas et al., 2009). Despite the clear connection of these Hsp60 mutants with impaired refolding activity, how these mutations and their defects in refolding activity contribute to the disease pathogenesis remains to be determined.

Besides Hsp60 mutations, abnormal expression level of Hsp60 has also been reported to associate with various diseases, which may also underscore the importance of unique localization pattern of Hsp60 as mentioned above. Hsp60 has been reported to be involved in inflammatory responses and immune reactions (Pockley, 2003). Therefore, the expression level of Hsp60 can potentially modulate these pathophysiological pathways. For example, the expression level of Hsp60 in skin allografts could modulate the host rejection toward the allografts where high level of expression leads to enhanced rejection in non-obese diabetic (NOD) mice (Birk et al., 1999). In this regard, Hsp60 has been shown to be able to function as an autoantigen and the Hsp60 autoimmunity can be modulated in NOD mice by subcutaneous injection of mouse Hsp60 peptides (Elias et al., 1990). This vaccination is protective against allograft rejection (Birk et al., 1999). Mechanistically, this Hsp60 vaccination strategy appears to involve a shift in the phenotype of the T cell response to self Hsp60 from a proinflammatory Th1 type of response to a Th2 regulatory type of response (Elias et al., 1997; Birk et al., 1999). The concept that endogenous Hsp60 can function as an autoantigen leading to production of anti-Hsp60 antibody has also been validated in humans. Patients with spondyloarthritis or periodontitis present higher tier of Hsp60 antibody than normal healthy volunteers (Tabeta et al., 2000; Hjelholt et al., 2013). However, human serum anti-Hsp60 level seems to be independent of predicting kidney allograft rejection (Trieb et al., 2000). The autoimmunity against Hsp60 may serve as a protective factor against the development of atherosclerosis upon aging (Wick, 2016; Zhong et al., 2016). The Hsp60's role as an autoantigen has also been illustrated in the development of a range of other autoimmune diseases including Hashimoto's thyroiditis (Marino Gammazza et al., 2014; Tonello et al., 2015), myasthenia gravis (Astarloa and Castrillo, 1996; Cappello et al., 2010; Marino Gammazza et al., 2012), inflammatory bowel diseases (Tomasello et al., 2011; Füst et al., 2012), chronic obstructive pulmonary diseases (COPD) (Cappello et al., 2011). The involvement of Hsp60 in these different autoimmune diseases is very interesting because one of the clinically used immunosuppressant mizoribine targets Hsp60 (see below).

Hsp60 is also implicated in the cell survival and apoptosis signaling pathways (Czarnecka et al., 2006), the balance of which is the key to the pathogenesis of cancers (Hanahan and Weinberg, 2011). Increased protein level of Hsp60 has been detected from both solid tumor tissues including breast (Bini et al., 1997; Desmetz et al., 2008), colon (Cappello et al., 2003a; He et al., 2007), cervix (Cappello et al., 2002; Hwang et al., 2009), prostate (Cappello et al., 2003b; Castilla et al., 2010), lung (Xu et al., 2011), ovary (Hjerpe et al., 2013), and liquid tumor samples including acute myeloid leukemia (AML) (Thomas et al., 2005). In many of the cases examined, higher expression is correlated with poorer prognosis (Thomas et al., 2005; Xu et al., 2011; Hjerpe et al., 2013). On the other hand, higher expression of Hsp60 was observed in early-stage ovarian cancer than advanced-stage in one other report (Schneider et al., 1999). As mentioned above, Hsp60 has been detected in blood circulation. A few studies have suggested that circulating Hsp60 protein level or the autoantibody against Hsp60 has potential value in early detection of colorectal cancer and breast cancer (He et al., 2007; Desmetz et al., 2008; Hamelin et al., 2011). If proven, this will add significantly to the field of cancer early detection. The extracellular Hsp60 does not seem to be present as a free form. Instead, several studies (Merendino et al., 2010; Campanella et al., 2012; Hayoun et al., 2012; Caruso Bavisotto et al., 2017) have shown that Hsp60 is packaged in exosomes, which are extracellular vesicles involved in intercellular communications (van Niel et al., 2018). The exosome-localized Hsp60 has been proposed to be actively secreted via endoplasmic reticulum-Golgi secretory pathway, which is inhibitable by brefeldin A or monensin (Campanella et al., 2012; Hayoun et al., 2012). The secreted Hsp60 was also found to be glycosylated in the secretory pathway (Hayoun et al., 2012). Interestingly, Hsp60 from normal cells does not seem to be secreted via this mechanism (Hayoun et al., 2012). Furthermore, it was recently found that a human lung-derived carcinoma cell line H292 treated with a histone deacetylase inhibitor vorinostat showed elevated Hsp60 level in exosomes (Campanella et al., 2016) although the potential clinical significance of this finding remains to be established.

Hsp60's exact role during carcinogenesis is not very clear (Cappello and Zummo, 2005) and it is possible that the altered expression in different cancers is due to its moonlighting functions outside mitochondria. In this regard, cytosolic Hsp60 has been shown to directly interact with the inhibitor of κB kinase (IKK) to promote TNFα-mediated activation of NF-κB-dependent gene transcription and survival of cancer cells (Chun et al., 2010). Hsp60 has also been observed to interact with β-catenin to enhance its transcription activity in the Wnt signaling pathway and promote cancer cell metastasis (Tsai et al., 2009). This interaction likely occurs in the cytosol instead of mitochondria.

## Hsp60 modulators

As described above, the Hsp60-Hsp10 chaperone complex is very important in maintaining mitochondrial homeostasis and plays a critical role in different diseases including autoimmune diseases and cancers. Developing small molecule modulators that can target Hsp60 is potentially useful as therapeutics in these disease areas (Nakamura and Minegishi, 2013; Cappello et al., 2014). In addition, such small molecule modulators can be powerful chemical tools to further elucidate the biological functions of Hsp60 in different contexts. Although numerous natural and synthetic compounds have been developed to target another chaperone protein Hsp90, relative few have been developed to target Hsp60 (Nakamura and Minegishi, 2013; Pace et al., 2013; Cappello et al., 2014; Radons, 2017). Most the Hsp60 inhibitors developed so far are derived from chemoproteomics studies of known bioactive compounds. The known Hsp60 inhibitors are either from natural products or synthetic compounds. Mechanistically, these inhibitors can be classified into two types. Type I inhibitors are found to block ATP binding and hydrolysis. Because the ATP-dependent conformational changes are affected, the Hsp60-Hsp10's refolding activity is inhibited by these inhibitors. Type II inhibitors include compounds that covalently react with certain cysteine residues in Hsp60. However, the details of these inhibitors' binding sites have not been revealed definitely. The following section will summarize the known Hsp60 modulators. We classify the inhibitors based on their sources of discovery, i.e., natural products or synthetic compounds.

### Natural products-based Hsp60 inhibitors

**Figure 1:**
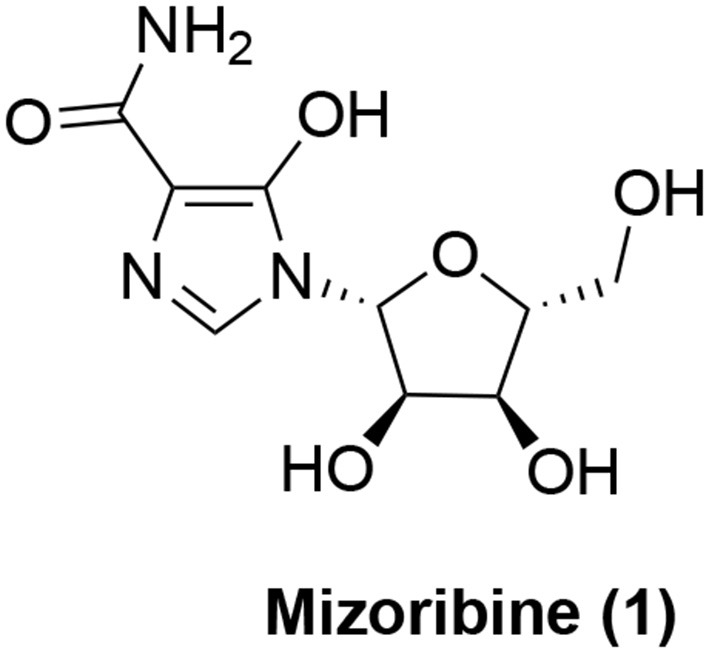
Chemical structure of mizoribine (**1**).

The search for small molecule inhibitors of Hsp60 started with natural products. The first small organic molecule to be known as an Hsp60 inhibitor is mizoribine (**1**, Figure 1). Mizoribine is an imidazole nucleoside antibiotics and was isolated from *Eupenicillium brefeldianum* (Mizuno et al., 1974). Mizoribine is devoid of anti-microbial activity, but has potent immunosuppressive activity (Mizuno et al., 1974) and has been used clinically after renal transplantation (Tajima et al., 1984). Its immunosuppressive activity is postulated to be related to mizoribine monophosphate derived from adenosine kinase reaction after cellular uptake. Mizoribine monophosphate inhibits inosine monophosphate (IMP) dehydrogenase and guanosine monophosphate (GMP) synthase resulting in depletion of intracellular GTP level to block T cell proliferation (Turka et al., 1991). In an effort to identify the direct binding proteins of mizoribine, an affinity reagent was prepared based on mizoribine and found that it bound to Hsp60 (Itoh et al., 1999). This direct binding led to inhibition of the chaperone activity of the Hsp60-Hsp10 complex. The ATPase activity of Hsp60 was also inhibited by mizoribine, which was accompanied with more stable association of Hsp10 with Hsp60 (Tanabe et al., 2012). Interestingly, the effect of mizoribine on the bacterial GroEL-GroES complex is much less pronounced (Tanabe et al., 2012), suggesting that selective targeting can be achieved even with the highly homologous proteins. As mentioned above, Hsp60 is also involved in autoimmunity, it is tentative to speculate that mizoribine's activity on the Hsp60-Hsp10 complex or Hsp60 alone may also contribute to its immunosuppressive effect although supplementing GTP could reverse mizoribine's immunosuppressive effect (Turka et al., 1991). In this respect, it is of note that mM concentrations of mizoribine are needed to inhibit Hsp60's activity (Tanabe et al., 2012) while the clinically achievable plasma concentrations of mizoribine were only ~30 μM (Honda et al., 2006). However, further medicinal chemistry optimization of mizoribine to improve its Hsp60-targeting activity has not been reported.

**Figure 2:**
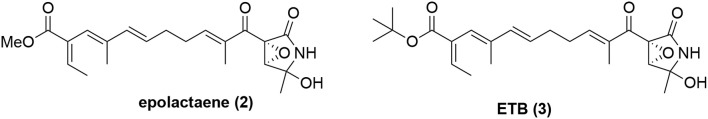
Chemical structures of epolactaene (**2**) and its *tert*-butyl ester ETB (**3**).

**Figure 3:**
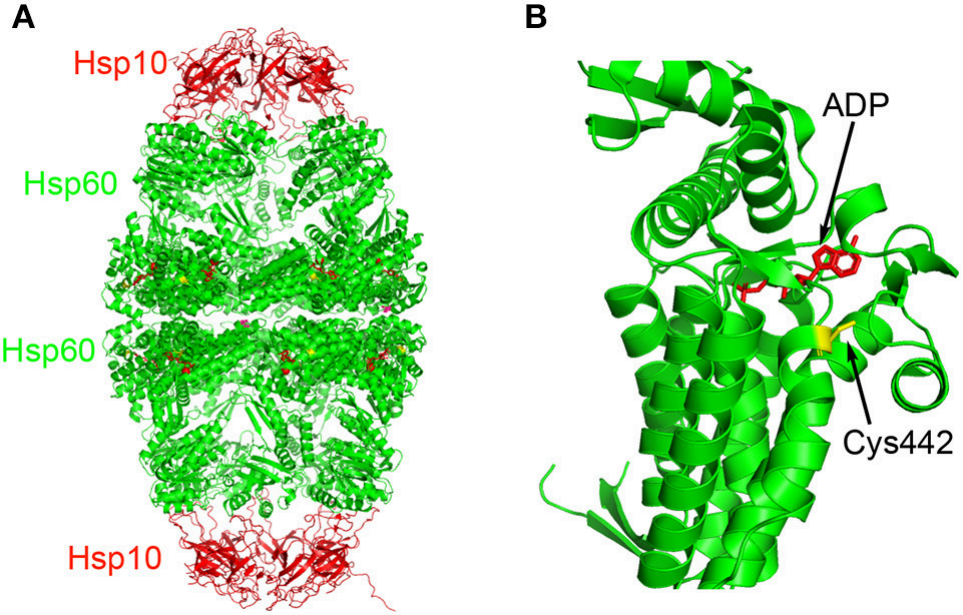
Binding site of ETB on Hsp60. **(A)** The Hsp60-Hsp10 complex (PDB: 4PJ1) is color-coded in red (Hsp10) and green (Hsp60) cartoons. The ADP molecules are presented in stick model in red and Cys442 residues are presented in stick model in yellow. **(B)** Close-up of the ADP binding pocket along with Cys442 in Hsp60.

Another natural product known to inhibit Hsp60 is epolactaene (**2**, Figure 2), which was originally isolated from the fungal strain *Penicillium* sp. BM 1689-P and was shown to be able to promote neurite outgrowth in SH-SY5Y cells (Kakeya et al., 1995). Its *tert*-butyl ester ETB (**3**, Figure 2) was shown to be as active as epolactaene (Nagumo et al., 2004). However, it had an unknown mechanism of action. To identify the direct molecular targets of ETB, a biotinylated ETB was synthesized for pulldown experiments (Nagumo et al., 2005). Mass spectrometry identification of the precipitated proteins identified that ETB bound to Hsp60. In a competition experiment, ETB was shown to selectively bind to Hsp60 without appreciable binding to other chaperone proteins including Hsp70 and Hsp90 (Nagumo et al., 2005). This binding interaction also led to inhibition of Hsp60-Hsp10's chaperoning activity. Further biochemical studies showed that ETB covalently reacted with Cys442 of Hsp60 (Nagumo et al., 2005). Mapping this residue to the recently solved X-ray crystal structure of human Hsp60-Hsp10 complex (Nisemblat et al., 2015) revealed that it is located at a site in close proximity to the ATP binding pocket (Figure 3), suggesting potential allosteric modulation. Although there are multiple reactive electrophilic centers in ETB, the α,β-unsaturated ketone is the most likely conjugation site for ETB based on additional structure-activity relationship (SAR) studies (Nagumo et al., 2004). Interestingly, ETB does not inhibit the ATPase activity of Hsp60 (Ban et al., 2010), suggesting that the covalent interaction between ETB and Cys442 may allosterically modulate Hsp60-Hsp10's chaperoning activity without interfering with its ATPase activity. While Cys442 modification does not modulate Hsp60's ATPase activity, Cys138 alkylation in GroEL significantly enhances its ATPase activity although these Cys residues are not conserved (Martin, 1998; Parnas et al., 2012). Despite the clear biochemical evidence to support that ETB targets Hsp60, it remains to be established how this binding and modulation of Hsp60 are linked to ETB's activity in promoting neurite outgrowth in SH-SY5Y cells.

**Figure 4:**
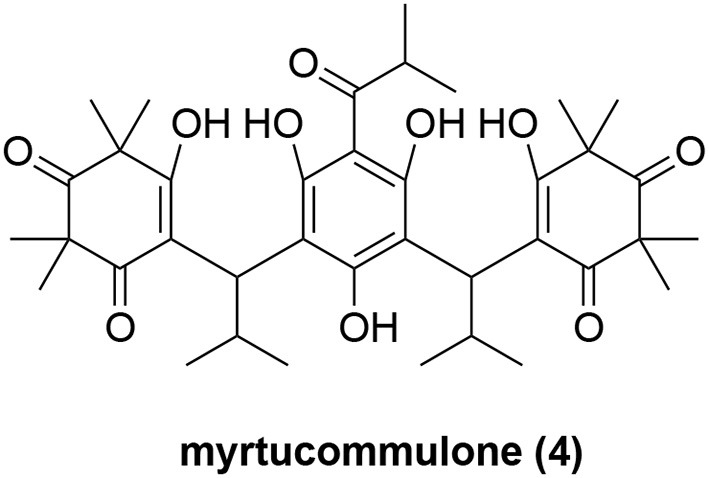
Chemical structure of myrtucommulone A (MC, **4**).

Recently, myrtucommulone A (MC, **4**, Figure 4) was identified as yet another natural product to inhibit Hsp60 (Wiechmann et al., 2017). MC is a non-prenylated acylphloroglucinol with multiple reported bioactivities, including antibacterial (Rotstein et al., 1974; Appendino et al., 2002), antioxidant (Rosa et al., 2003), anti-inflammatory (Feisst et al., 2005; Rossi et al., 2009), and anti-tumor properties (Tretiakova et al., 2008; Grandjenette et al., 2015; Izgi et al., 2015). MC was found to act on isolated mitochondria from human leukemia cells, and to affect mitochondrial functions at submicromolar concentrations, including loss of mitochondrial membrane potential (Δψm), reduction of mitochondrial viability and inhibition of mitochondrial ATP synthesis (Wiechmann et al., 2015). But the exact molecular targets of MC within mitochondria were unknown. Toward this end, MC was immobilized onto sepharose resin to pulldown cellular proteins that might bind to MC (Wiechmann et al., 2017). Although multiple protein bands were observed to bind to MC, Hsp60 was the most prominent one. Further biochemical validation experiments showed that Hsp60 is a direct mitochondrial protein target of MC and MC inhibited the refolding activity of the Hsp60-Hsp10 complex. Moreover, the authors also proposed that Hsp60 is likely to protect the mitochondrial proteins Lon protease-like protein (LONP) and leucine-rich protein 130 (LRP130) against heat-shock-induced aggregation because they are both significantly influenced by MC (Wiechmann et al., 2017). While MC clearly can modulate Hsp60's activity, it was reported that MC also targets microsomal prostaglandin E2 synthase 1 (mPGES-1) and 5-lipoxygenase (5-LOX) (Feisst et al., 2005; Koeberle et al., 2009) to affect arachidonic acid metabolism. To further probe the Hsp60 biology with MC and assess its therapeutic potential, it will be critical to develop MC analogs that are devoid of these other biological activities. Its high hydrophobicity (cLogP = 5.5) and presence of multiple redox-active groups pose nontrivial challenges to develop improved analogs.

**Figure 5:**
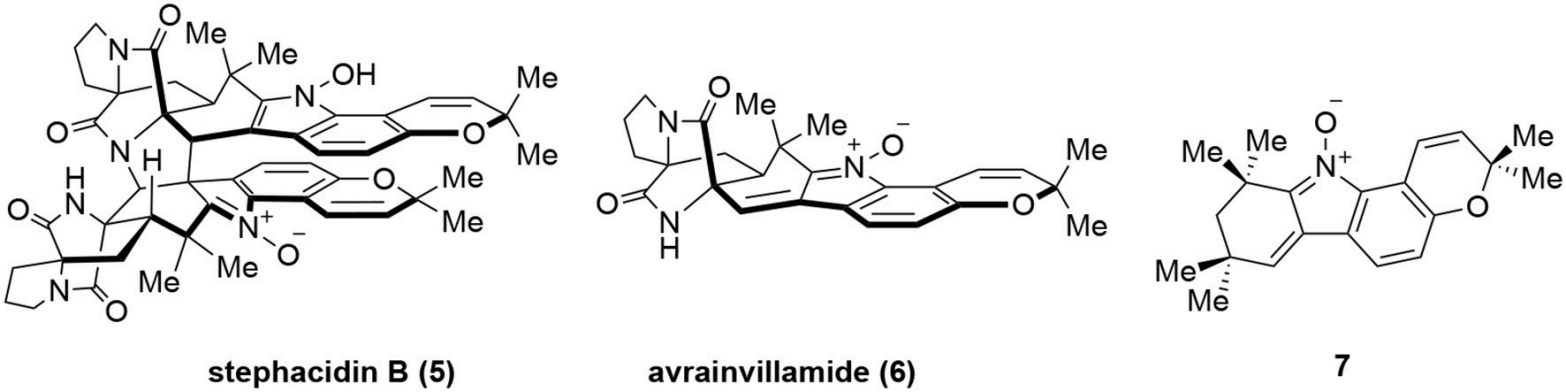
Chemical structures Stephacidin B (**5**), avrainvillamide (**6**), and a simplified analog **7**.

In addition to the above-mentioned natural products, several other natural products were also reported to interact with Hsp60 although stringent validation data are lacking. Stephacidin B (**5**, Figure 5) is a natural product isolated from *Aspergillus ochraceus* WC76466 (Qian-Cutrone et al., 2002) while avrainvillamide (**6**) was isolated from *Aspergillus* sp. CNC358 (Fenical et al., 2000). Both of them showed potent *in vitro* anticancer activities. It was found that dimeric stephacidin B (**5**) was converted into monomeric **6** in tissue culture media and suggested that **6** was the actual active species during cellular experiments (Wulff et al., 2007). Indeed, after correcting molar equivalent, **5** and **6** had almost identical activity in the cellular assays. Furthermore, a simplified undimerizable analog **7** also presented anticancer activity albeit with reduced potency (Wulff et al., 2007). To identify the potential binding targets of **7**, a biotinylated derivative of **7** was prepared to pulldown its targets. This identified Hsp60 as one of the putative targets for **7** and perhaps for **5** and **6** (Wulff et al., 2007). However, further validation studies have yet to be performed and whether these complex natural products are *bona fide* Hsp60 modulators remains to be established.

### Hsp60 inhibitors originated from synthetic sources

**Figure 6:**
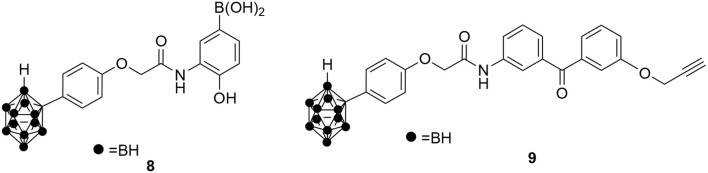
Chemical structures of *o*-carboranylphenoxyacetanilide **8** and its clickable photoaffinity probe **9**.

Besides the natural products identified above as potential Hsp60 modulators, quite a few synthetic molecules have also been discovered to be able to modulate Hsp60. In 2010, *o*-carboranylphenoxyacetanilide **8** (Figure 6) was identified as a hypoxia-inducible factor 1 alpha (HIF-1α) inhibitor using a transcription reporter assay (Shimizu et al., 2010). HIF-1α is often stabilized and activated in cancer tissues and inhibiting HIF-1α's transcription activity has great potential to develop novel cancer therapeutics (Semenza, 2003). To identify the direct molecular targets of *o*-carboranylphenoxyacetanilide, a clickable photoaffinity probe **9** was designed and synthesized based on *o*-carboranylphenoxyacetanilide (Figure 6; Ban et al., 2010). The benzophenone moiety in probe **9** could covalently crosslink with the direct target proteins upon ultraviolet (UV) irradiation. Then the propargyl group in probe **9** can be clicked with Alexa Fluor 488 azide to visualize the proteins bound to the probe. Using this strategy, it was found that *o*-carboranylphenoxyacetanilide bound to Hsp60 (Ban et al., 2010). Under the same conditions, the probe **9** did not label other heat shock proteins including Hsp90 and Hsp70 (Ban et al., 2010), suggesting specific binding to Hsp60. Further validation studies showed that compound **8** inhibited Hsp60-Hsp10's refolding activity and Hsp60's ATPase activity. Importantly, Hsp60 was found to interact with HIF-1α (Ban et al., 2010), suggesting that binding of **8** to Hsp60 can be implicated in inhibition of HIF-1α-mediated gene transcription. Since HIF-1α is a nuclear located protein, it is possible that the interaction between Hsp60 and HIF-1α occurs inside the nucleus, but other possibilities cannot be excluded.

**Figure 7:**
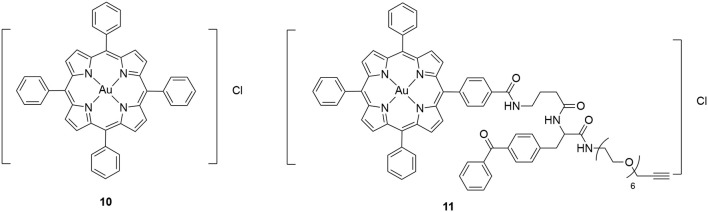
Chemical structure of gold (III) porphyrin [Au(TPP)]Cl (**10**) and its clickable photoaffinity probe **11**.

The other class of synthetic compounds identified to inhibit Hsp60 is gold (III) porphyrin complexes. Many gold (III) complexes were shown to possess anticancer activity against different cancer cell lines (Nobili et al., 2010). However, their therapeutic potential was limited due to their instability under physiological conditions. This limitation has been overcome by synthesizing gold (III) complexes with strong donor ligands, which are much more stable under physiological conditions and also presented significant anticancer activities (Lease et al., 2013; Teo et al., 2014). A prototype gold (III) complex [Au(TPP)Cl] (**10**) is shown in Figure 7. One of the major challenges to move these gold (III) complexes forward is our limited understanding of their mechanism of action. As a first step toward this challenge, the binding protein targets of the gold (III) complexes need to be identified. A chemoproteomics strategy was designed to identify the binding targets of **10** using a clickable photoaffinity probe **11** (Figure 7; Hu et al., 2016). This target identification strategy is similar to that used for *o*-carboranylphenoxyacetanilide. Through this strategy, Hsp60 was identified as a direct molecular target of **10**. Further biochemical and cellular studies using saturation-transfer difference-nuclear magnetic resonance (STD-NMR) and cellular thermal shift assays demonstrated that **10** engaged interaction with Hsp60 both *in vitro* and in cells (Hu et al., 2016). It was further found that **10** inhibited the refolding activity of the Hsp60-Hsp10 complex. Additional SAR studies demonstrated that both the gold (III) ion and porphyrin ligand are necessary for the inhibitory activity (Hu et al., 2016). It is unclear if the ATPase activity of Hsp60 or other chaperone proteins was inhibited by **10** and its derivatives. It is speculated that the gold (III) ion may interact with Hsp60 electrophilically and the porphyrin ligand may bind to Hsp60 through hydrophobic interactions (Hu et al., 2016). However, the detailed mechanism of action of these gold (III) complexes remain to be elucidated.

## Conclusions and outstanding questions

Since the initial discovery of Hsp60 as the mitochondrial molecular chaperone, many studies have shown that it is also localized outside mitochondria with perhaps both chaperoning and non-chaperoning activities. Therefore, it is not surprising that many different disease states especially autoimmune diseases and cancers have presented altered expression level of Hsp60. This presents a great opportunity to develop potential therapeutics by targeting Hsp60. Quite a few different small molecule modulators of Hsp60 have been identified. These include both natural products and synthetic molecules. It is striking that these different small molecules have no common structural motifs or pharmacophores, yet they all modulate Hsp60's activity. It will be critical to understand how these different inhibitors can all interact with the Hsp60-Hsp10 complex. Some differences have already been noticed among different inhibitors. While all of the identified inhibitors can inhibit the refolding activity, not all of them inhibit the ATPase activity. It is possible that some of these reported inhibitors, especially the hydrophobic ones, may also inhibit the spontaneous folding of the substrate proteins independent of Hsp60. Future characterization of the inhibitors should include this type of critical controls to determine the extent of Hsp60 involvement. The recent breakthrough in determining the X-ray crystal structure of the human Hsp60-Hsp10 complex (Nisemblat et al., 2015) shall facilitate our understanding of how the inhibitors interact with Hsp60. Given the structural diversity of the reported Hsp60 modulators, both orthosteric and allosteric modulation mechanisms are possible. As a consequence, different inhibitors may distinctly affect the Hsp60-Hsp10 dynamics and the individual steps in the folding cycle (Weiss et al., 2016). Mechanistically, it will also be critical to elucidate how the inhibitors binding to Hsp60 can result in the distinct phenotype of the inhibitors (e.g., anticancer activity) because most of these Hsp60 modulations were discovered after the initial findings of the bioactivities of the inhibitors through chemoproteomics approaches. These studies will in turn inform their future development into potential therapeutics.

While mizoribine was shown to be selective in targeting human Hsp60-Hsp10 vs. bacterial GroEL-GroES (Tanabe et al., 2012), a large number of small molecules were identified as GroEL-GroES inhibitors from a high-throughput screening of ~700,000 compounds (Johnson et al., 2014; Abdeen et al., 2016a). Interestingly, most of these inhibitors are more selective against GroEL-GroES vs. Hsp60-Hsp10 (Abdeen et al., 2016a,b). Some of these may even potentially inhibit the Hsp60-Hsp10 complex from *Trypanosoma brucei* (Abdeen et al., 2016b) to treat African sleeping sickness. These results suggest that selectively targeting one protein complex is feasible. Ongoing extensive studies are attempting to resolve if the Hsp60 protein complexes in different subcellular compartments have unique activities or oligomeric equilibria (Vilasi et al., 2017), which may offer additional opportunities to develop small molecules to target one complex over the others. For example, can we selectively target one Hsp60 complex that is more disease relevant than the mitochondrial Hsp60-Hsp10 complex that is essential for normal mitochondrial homeostasis?

As we expect that more inhibitors are being developed and more mechanistic details are being elucidated, targeting Hsp60 can be a powerful strategy to develop therapeutics in multiple indications. When assessing these inhibitors, it is critical that appropriate controls are included to ensure that the inhibitors are indeed targeting Hsp60 instead of other components in the assay system (e.g., spontaneous folding or other enzymes included for coupled biochemical assays). In this regard, both substrates that can refold spontaneously (e.g., green fluorescent protein and dihydrofolate reductase) and substrates whose refolding depends on Hsp60 (e.g., rhodanese and malate dehydrogenase) should be used. Refolding reactions in the absence of the chaperonin complex should also be included to evaluate potential inhibitors. With these proper controls, one can ascertain that the inhibitors indeed directly target Hsp60-dependent activity and even possibly tease out the exact step that the inhibitors actually act on in this multi-step folding process. The potencies of the current inhibitor are in general low (μM to mM) and more potent inhibitors (preferably low to high nM range) need to be developed for further studies. It is tempting to speculate that small molecules that can enhance the Hsp60's chaperone activity may provide a novel opportunity to treat neurodegenerative disorders where Hsp60 mutations cause defective chaperones. Molecules possessing this activity have not been reported yet, but allosteric modulators to enhance Hsp60's refolding efficiency are likely to be discovered. On the other hand, the small molecules that can inhibit Hsp60's activities including refolding activity, ATPase activity and perhaps other moonlighting activities would be great starting points for new therapeutics in inflammatory diseases, autoimmune diseases and various cancers. As we better characterize the moonlighting functions of Hsp60 in the future, it will also be critical to assess the correlation between different compounds' inhibitory potency in Hsp60 refolding and the moonlighting function in question. Such studies will further shed insights into the biochemical properties of Hsp60 in different subcellular compartments. Most of the inhibitors developed so far possess different degrees of anticancer activities. When deciding the future preclinical and clinical applications of these small molecule modulators, it will be critical to determine the selectivity profiles of the inhibitors and to what degree the mitochondrial homeostasis will be altered by the small molecules and how this effect will confer potential deleterious side effects.

## Author contributions

All authors listed have made a substantial, direct and intellectual contribution to the work, and approved it for publication.

### Conflict of interest statement

The authors declare that the research was conducted in the absence of any commercial or financial relationships that could be construed as a potential conflict of interest.
